# Eco-Friendly Incorporation of Crumb Rubber and Waste Bagasse Ash in Bituminous Concrete Mix

**DOI:** 10.3390/ma15072509

**Published:** 2022-03-29

**Authors:** Sheraz Ullah, Muhammad Izhar Shah, Muwaffaq Alqurashi, Muhammad Faisal Javed, Osama Dawood, Fahid Aslam, Muhammad Atiq Ur Rehman Tariq, Enas E. Hussain

**Affiliations:** 1 Department of Civil Engineering, COMSATS University Islamabad, Abbottabad Campus, Abbottabad 22060, Pakistan; sherazullahwazir@gmail.com (S.U.); arbabf1@gmail.com (M.F.J.); osamaa.daud@gmail.com (O.D.); 2 Department of Civil Engineering, College of Engineering, Taif University, P.O. Box 11099, Taif 21944, Saudi Arabia; m.gourashi@tu.edu.sa; 3 Department of Civil Engineering, College of Engineering in Al-Kharj, Prince Sattam Bin Abdulaziz University, Al-Kharj 11942, Saudi Arabia; f.aslam@psau.edu.sa; 4 College of Engineering and Science, Victoria University, Melbourne, VIC 8001, Australia; atiq.tariq@yahoo.com; 5 National Water Research Center, P.O. Box 74, Shubra El-Kheima 13411, Egypt

**Keywords:** waste utilization, bituminous concrete, experimental testing, indirect tensile strength, Marshall stability

## Abstract

The consumption of waste materials in the construction sector is a sustainable approach that helps in reducing the environmental pollution and decreases the construction cost. The present research work emphasizes the mechanical properties of bituminous concrete mix prepared with crumb rubber (CR) and waste sugarcane bagasse ash (SCBA). For the preparation of bituminous concrete mix specimens with CR and SCBA, the effective bitumen content was determined using the Marshall Mix design method. A total of 15 bituminous concrete mix specimens with 4%, 4.5%, 5%, 5.5% and 6% of bitumen content were prepared, and the effective bitumen content turned out to be 4.7%. The effect of five different CR samples of 2%, 4%, 6%, 8% and 10% by weight of total mix and SCBA samples of 25%, 50%, 75% and 100% by weight of filler were investigated on the performance of bituminous concrete. A total of 180 samples with different percentages of CR and SCBA were tested for indirect tensile strength (ITS) and Marshall Stability, and the results were compared with conventional bituminous concrete mix. It was observed that the stability values rose with an increase in CR percentage up to 6%, while the flow values rose as the percentage of SCBA increased in the mix. Maximum ITS results were observed at 4% CR and 25% SCBA replacement levels. However, a decrease in stability and ITS result was observed as the percentages of CR and SCBA increased beyond 4% and 25%, respectively. We concluded that the optimum CR and SCBA content of 4% and 25%, respectively, can be effectively used as a sustainable alternative in bituminous concrete mix.

## 1. Introduction

The transportation system is a primary pillar for linking cities all over the world. It consists of infrastructure such as railroads, highways, and bridges that allow people and goods to travel safely and efficiently inside and between communities [1]. The roadway is an essential part of the transport infrastructure, and engineers must recognize the safety and economic needs of road users. Currently, the number of vehicles is quickly expanding, resulting in tire waste, which is a serious environmental issue. Similarly, an increase in traffic loading and high tire pressure is causing degradation to the road network. Every year, approximately 1.5 billion tires are created around the world, with 1000 million tires reaching the end of their secondary life [2,3]. When used tires are destroyed and crushed, crumb rubber (CR) is produced. At present, about 60% of waste tires are disposed of in urban and rural areas, which results in environmental pollution [4]. Numerous research studies have been conducted on utilizing the waste and by-products in the construction industry. The successful incorporation of waste in cement and bituminous concrete will reduce environmental pollution, provide a cost-effective solution and ultimately enhance the performance of the structures being modified [5,6]. Examples of waste used as a modifier in bituminous concrete and cement concrete are coal fly ash, rubber, sugarcane fiber, ground granulated blast furnace slag, polyethylene terephthalate, alkali activated slag and wood bottom ash [3,7,8,9,10]. Sugarcane fiber (bagasse) is debris left behind after sugarcane is crushed and juice is extracted, and sugarcane bagasse ash (SCBA) is formed when bagasse is burned. Inappropriate waste disposal of bagasse ash can create a negative impact on the ecosystem near sugar refineries [11,12,13].

Bitumen is used to bind the aggregate to create asphalt concrete by covering and coating the aggregate. However, it has a low water resistance [14,15]. In general, bitumen binder quality influences the pavement performance. It is well-known that typical bitumen has a restricted range of rheological behavior and durability, which is insufficient to withstand pavement distresses [16]. Roads may lose their strong attributes because of increased demand, making them more susceptible to persistent distresses and failure. Improving the rheological qualities of conventional bitumen by mixing it with crumb rubber is a typical way to improve its quality [17,18]. The mixtures are created without any major interaction time between bitumen and CR, and the reaction between the two is regarded as minimal. However, the utilization of CR modified binders in pavement construction continues as several laboratory and field tests adequately demonstrated that asphalt mixes with CR improves the performance of the asphalt pavement [19,20,21,22,23,24,25,26,27,28]. The results indicate that CR can be used to make sustainable HMA mixes for use in flexible pavements handling severe traffic loads on highways, with numerous environmental advantages [29]. The CR-modified bituminous binders had stronger viscosity and temperature resistance than the conventional bituminous binders [29]. In considering the rheological properties, penetration, softening point and specific gravity, the CR modifier gives better mechanical properties as compared to conventional asphalt concrete [30,31].

Li et al., (2018) [32] reported that the California Bearing Ratio increased with the inclusion of fine rubber content; therefore, CR can be reprocessed as a waste material for the base and subbase layers of the pavement. Navarro et al., (2005) [33] reported in their study that the storage stability of rubber-modified bitumen improved at 180 °C with the increase in the rubber concentration. The small particle size of the CR interacts with the binder more rapidly, enabling some alteration of the warmed bitumen [34,35,36]. Xiao et al., (2009) [37] conducted a study by adding CR to asphalt concrete and reported an increase in the voids in mineral aggregate and improved rutting resistance of the asphalt mix irrespective of the type and CR size. Similarly, the literature study showed that SCBA has been used in various studies in the production of cement concrete and asphalt concrete [38,39,40,41,42,43]. Zainudin et al., (2016) [3] reported that the SCBA modified mixes increase the resilient modulus, stability and flow of the asphalt concrete by 17.4%, 0.6% and 4.9 %, respectively, as compared to conventional hot mix asphalt (HMA). SCBA as a filler in HMA helps in reducing the need for cement fillers while providing a positive net worth and eliminating present wastage [44]. One ton of sugarcane yields around 250–270 kg of bagasse [38,45]. It is also know that that SCBA in flexible pavement can enhance the physical and rheological properties of bitumen based on chemical analysis [1,3]. Zia et al., (2021) [46] used SCBA as a filler in the production of modified HMA. The results showed a considerable increase in the performance of modified HMA as compared to conventional HMA.

It is evident from the literature survey that performance of the asphalt concrete in terms of stability and strength varies by using different dosages of CR and SCBA. To the authors’ best knowledge, the literature studies lack a comprehensive investigation on assessing the combination of CR and SCBA in asphalt concrete. Therefore, the present study is devoted to incorporating a blend of CR and SCBA in the production of eco-friendly asphalt concrete. A total of 180 asphalt concrete samples of 4 in diameter were prepared in the laboratory with different dosages of CR and SCBA. The Marshall stability, Marshall flow and indirect tensile properties of the specimens were investigated in the laboratory. Both the CR and SCBA are the by-products of different industries and place a burden on the environment that causes environmental issues. The present study aimed at minimizing the environmental pollution by utilizing the wastes SCBA and CR in flexible pavement.

## 2. Materials and Methods

### 2.1. Aggregates

**Figure 1:**
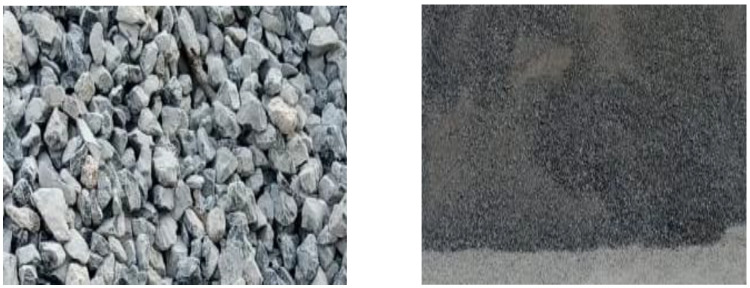
Coarse aggregates used in the study.

Aggregates used in this research were acquired from Ghumavan Crush Plant Thandiani Abbottabad, Pakistan. The selection of aggregates was based on their usage in wearing course in the production of asphalt concrete as shown in Figure 1. The properties of aggregates were evaluated employing specific gravity, Los Angeles abrasion, impact value, water absorption, and flakiness and elongation index, which resulted in selecting suitable aggregates for use in HMA. Table 1 illustrates the results of the above-mentioned tests, which show that the selected aggregates qualify for the ASTM standard criteria.

### 2.2. Bitumen

**Figure 2:**
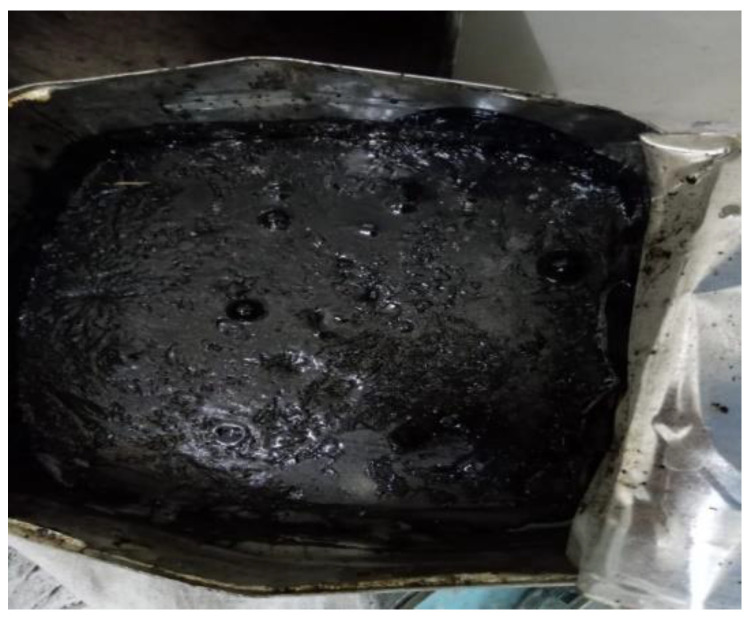
80/100 grade of bitumen used in the study.

The present study used 80/100 grade bitumen in accordance with the climatic conditions of the study region Abbottabad, Pakistan. The 80/100 grade bitumen was supplied and transported from Rawalpindi, Pakistan, as shown in Figure 2. Various tests were performed on bitumen to assess its properties, i.e., penetration, ductility, flash and fire point and softening point, and the results are illustrated in Table 2.

### 2.3. Crumb Rubber

**Figure 3:**
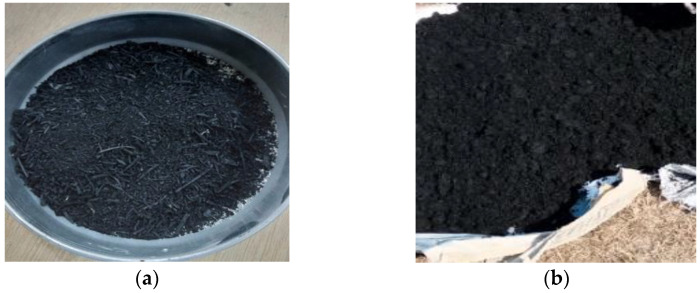
Waste materials used in this study. (**a**) Crumb Rubber (CR). (**b**) Sugarcane bagasse Ash.

The waste crumb rubber (CR) was acquired from Peshawar, Pakistan, in a ground form as depicted in Figure 3a. The CR was dark black in color and a bulk density of CR was found to be 301.5 kg/m^3^. The size of the CR ranged from 1–3 mm and was used as an additive from 0 to 10% by weight of the total mix (1200 gm) with an increment of 2% [56,57]. The complete chemical composition of the used CR is given in Table 3. Following the standard procedure, the CR was added to the bituminous mixtures using dry process in untreated form [35,58].

### 2.4. Sugarcane Bagasse Ash

Sugarcane bagasse ash (SCBA) is a waste from the sugar industry that is used as a fuel in the same industry. The resulting ash otherwise remains a problem and causes a reduction in land filling areas. In our study, the collected waste SCBA was subjected to grinding to make it compatible with filler material in the mix. Grinding was carried out in a ball mill machine to reduce the particle size until it passes from sieve # 8 and retained on sieve # 200. Table 4 shows the chemical composition of SCBA. The grinded SCBA was then used as a partial replacement of filler material in the mix ranging from 0–100%, with an increment of 25% by weight. Figure 3b shows the collected waste SCBA from sugar industry.

### 2.5. Mix Design for Hot Mix Asphlat (HMA)

In the present research, we followed the standard specifications of National Highway Authority (NHA) Pakistan’s, class “A” materials selection criteria. Table 5 and Table 6 respectively illustrate the NHA class “A” selection criteria and specification for Marshall Parameters such as Marshall stability and flow.

**Figure 4:**
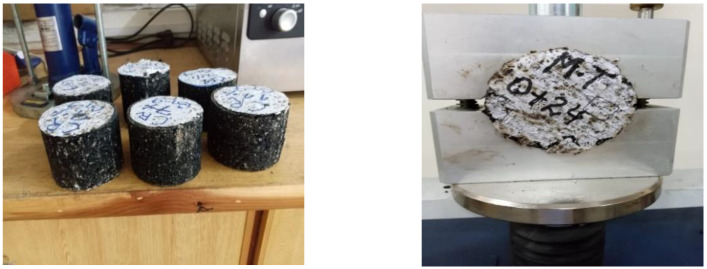
Asphalt concrete samples for Marshall stability testing.

For Marshall mix specimens preparation, the mixing temperature was increased gradually to 165 °C with bitumen content of 4%, 4.5%, 5%, 5.5% and 6% by sample weight at different stages [3,60]. A total of 15 asphalt concrete samples of 4 in diameter were prepared with 3 specimens for each bitumen percentage. Once the aggregate and bitumen were mixed carefully, the heated samples were placed in a mold and compacted instantly in the compactor. We applied 75 blows by the compactor on each side (top and bottom). Figure 4 shows the prepared asphalt concrete specimens for Marshall test. After the preparation of Marshall Mix samples, numerous tests were carried out on the prepared samples. These tests, namely Marshall stability and flow values, theoretical maximum specific gravity of the mix (Gmm) and bulk specific gravity of the mix (Gmb), were used assess the properties of bituminous mix and opt for the mix of desired properties. Gmm and Gmb were performed to calculate the volumetric parameters of Marshall Mix samples to assess the quality of the mix. Effective bitumen content (EBC) was calculated using Marshall stability and flow values on the freshly made conventional samples following the NHA class “A” standard specifications. The effective bitumen percentage was obtained from the tests carried out on the conventional samples prepared.

## 3. Results

### 3.1. Effective Bitumen Content

**Figure 5:**
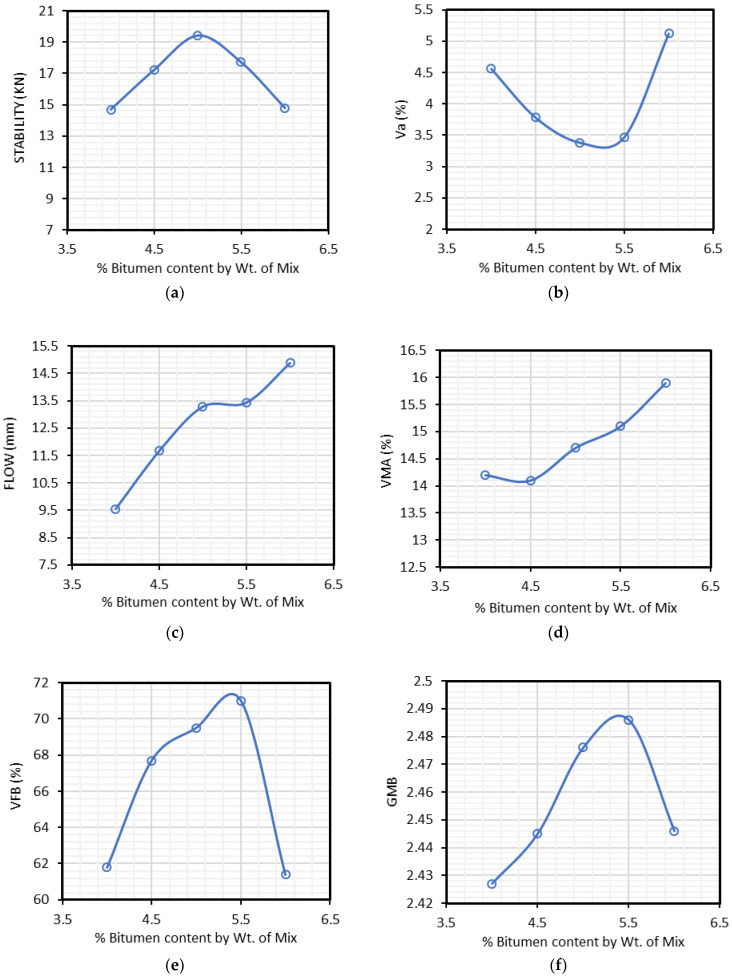
Effective bitumen content (EBC) calculation using the bitumen content in the mix versus (**a**) Marshall tability; (**b**) Air Voids (Va); (**c**) Marshall flow; (**d**) Voids in Mineral Aggregate (VMA); (**e**) Voids Filled by Bitumen (VFB); (**f**) Bulk Specific Gravity (Gmb).

The effective bitumen content was determined based on Marshall stability and flow of the samples (Figure 4) prepared according to ASTM D6927-15 standards [61]. Figure 5 presents the results of Marshall stability, Air voids, Marshall flow values, VMA, VFB, Gmb and the air voids. The mean value of bitumen content at maximum Marshall stability, Gmb, and the bitumen content at the median of the allowed percentage of air voids was calculated to obtain effective bitumen content. These values were found to be 4.5%, 4.5% and 5%, respectively. The EBC was determined to be at 4.7% by sample weight and the result was counter-checked with specification limits as described by NHA. Table 7 shows the calculated EBC of the mix.

### 3.2. Assessment of Mechanical Properties

After determination of Marshall volumetric properties and EBC, numerous samples of asphalt concrete were prepared in the lab with and without the addition of CR and SCBA. The CR was used from 0%, 2%, 4%, 6%, 8% and 10% of the weight of the total mix. SCBA was used as 0%, 25%, 50%, 75% and 100% by weight of the filler in the mix. Samples for Marshall stability and flow values, and indirect tensile strength (ITS) were cast with the abovementioned percentages of CR and SCBA. Three samples were prepared for each mix and then mean values were considered. The results of ITS, stability and flow values for conventional and modified asphalt concrete specimens are discussed in the subsequent sections.

### 3.3. Indirect Tensile Strength (ITS) for Modified Asphalt Mix

**Figure 6:**
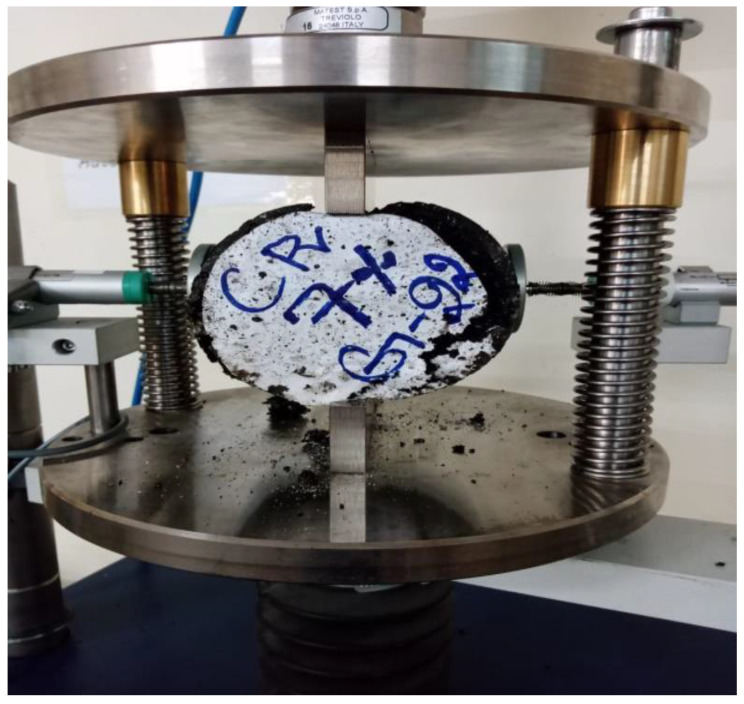
Testing of the asphalt concrete specimens for Indirect Tensile Strength (ITS).

**Figure 7:**
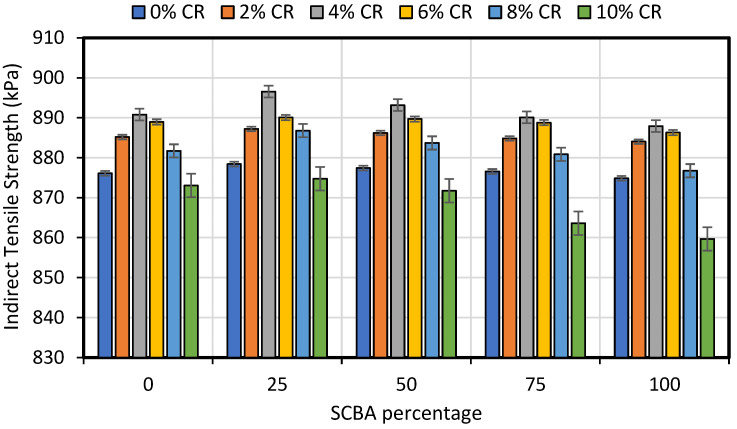
Effect of additions of CR and SCBA on split tensile strength of asphalt concrete.

ITS test was conducted to determine the resistance of asphalt concrete mix to cracking with various dosages of CR and SCBA. ITS test was conducted as per the standard protocols of ASTM D 6931 [62] shown in Figure 6. The results of the ITS test are presented in Figure 7, which shows that the ITS values increase as the percentage of CR and SCBA increases. For all the asphalt mixes with 0%, 25%, 50%, 75% and 100% SCBA, the maximum ITS value was observed at 4% CR. In addition, the maximum ITS was observed at 25% SCBA replacement. The lowest ITS value was observed for 10% CR replacement.

### 3.4. Marshall Stability and Flow for Modified Asphalt Mix 

**Figure 8:**
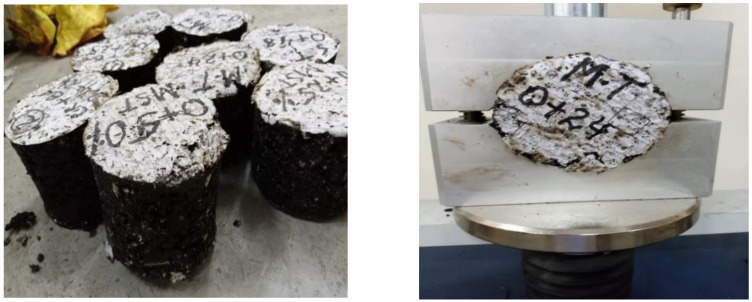
Asphalt concrete specimens prepared and tested for Marshall stability and flow.

As discussed in the previous section, the SCBA was used as 0%, 25%, 50%, 75% and 100% by weight of the filler in the mix, and the CR content of 0%, 2%, 4%, 6%, 8% and 10% was utilized by weight of the total mix [44,63]. Figure 8 shows the asphalt specimens prepared and tested for Marshall stability and flow. The literature survey revealed that CR melts at 105.5 °C, and therefore the mixing was again done at a temperature that gradually rose to 165 °C [64].

**Figure 9:**
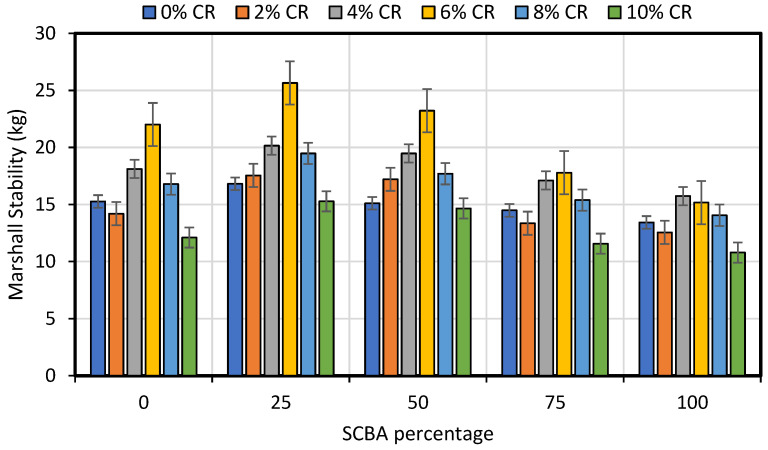
Effect of additions of CR and SCBA on stability of asphalt concrete.

**Figure 10:**
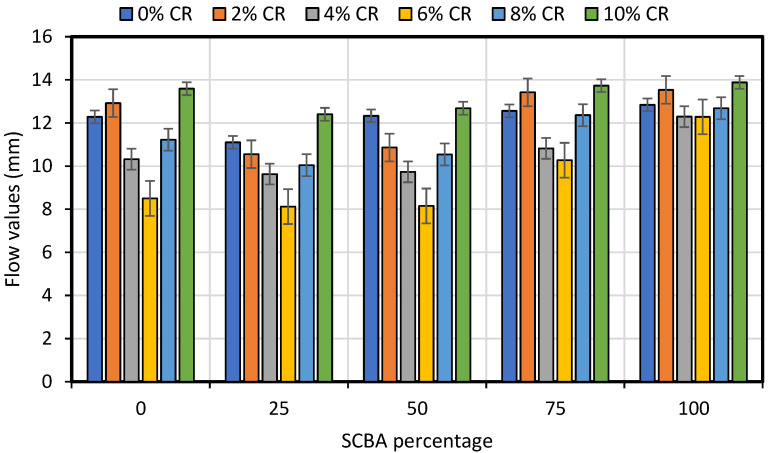
Effect of additions of CR and SCBA on flow of asphalt concrete.

Figure 9 and Figure 10 graphically show the result of stability and flow values for conventional, CR and SCBA-modified asphalt concrete samples. The results revealed that the stability values gradually increase with the addition of CR up to 6% and consistent decrease was observed thereafter. The similar trend of result can be observed for SCBA where maximum stability values were found for 25% SCBA replacement. Conclusively, maximum stability values were achieved at 6% CR and 25% SCBA replacement in the asphalt concrete mixes. As for the Marshall flow values, the minimum flow values were observed for asphalt concrete mix modified with 6% CR and 25% SCBA. The maximum flow values were found for the mixes with 10% CR and 100% SCBA. Evidently, the results revealed maximum stability and minimum flow results at 6% CR and 25% SCBA.

## 4. Conclusions

The present study evaluated the effectiveness of crumb rubber (CR) and waste sugarcane bagasse ash (SCBA) in the production of sustainable hot mix asphalt (HMA). The CR was incorporated in the asphalt mixes from 0 to 10% by weight of total asphalt mix with 2% increment. The filler material in the mix was replaced with SCBA from 0%, 25%, 50%, 75% and 100% by weight of the filler weight. A total of 180 asphalt concrete samples with 90 for Marshall stability and flow values and 90 samples for indirect tensile strength (ITS) test were prepared by replacing CR and SCBA by the abovementioned percentages. Based on experimental results and analysis, the following conclusions were drawn:

- By increasing the percentage of CR, the stability increased up to 6% and then gradually decreased up to 10% replacement level. The flow values gradually decreased as the percentage of CR increased up to 6% and then a consistent increase was observed up to 10%. Similarly, at 25% SCBA replacement level, maximum stability and minimum flow values were achieved.
- Maximum ITS results were achieved at 4% CR as an additive and 25% SCBA as a filler material in Hot Mix Asphalt. Hence it was concluded that increasing the percentage of SCBA beyond 25% flow values increases but stability decreases.
- The effective dosages of CR and SCBA in asphalt concrete specimens were observed to be 6% and 25% by weight of the total mix and weight of the filler in the mix, respectively.

The present study was mainly focused on utilizing wastes in asphalt concrete by considering a certain set of experimental tests. It is recommended that further study be conducted by utilizing different percentages of CR and SCBA. Additional experimental tests should be performed to better understand the viability of the waste materials in asphalt concrete. Moreover, the wet method of adding CR to asphalt concrete should be considered for further investigation.

## Figures and Tables

**Table 1:** Properties of the aggregates with standard specifications.

Aggregate Tests	ASTM Standards	Specification Limits	Results
Specific gravity	ASTM C127 [47]	–	2.68
Los Angeles abrasion (%)	ASTM C131 [48]	<30%	20.43
Impact value (%)	ASTM D 5874-95 [49]	<30%	15.12
Water absorption (%)	ASTM C128 [47]	–	0.5
Flakiness (%)	ASTM D 4791-99 [50]	10%	7.45
Elongation (%)	ASTM D 4791-99 [50]	10%	6.34

**Table 2:** Properties of the bitumen with standard specifications.

Bitumen Tests	ASTM Standards	Specification Limits	Results
Softening Point	ASTM D36-95 [51]	48–56 °C	53 °C
Ductility	ASTM D113-86 [52]	min 100 cm	109 cm
Penetration	ASTM D5-97 [53]	60–70 MM	66.53 mm
Flash Point	ASTM D92 [54]	min 232 °C	279 °C
Fire Point	ASTM D92 [54]	min 242 °C	284 °C
Specific Gravity	ASTM D70 [55]	1.01–1.06	1.04

**Table 3:** Chemical composition of crumb rubber [59].

Material	Weight (%)
Rubber Hydrocarbons	45.2
Carbon black	25.8
Acetone Extract	14.2
Isoprene	12.1
Water	0.8
Ash content	0.9
Fiber content	0.5
Metal Content	0.08
Others	0.42

**Table 4:** Oxides composition of sugarcane bagasse ash (SCBA).

Oxides, wt%	
SiO_2_	66.70
Al_2_O_3_	9.24
Fe_2_O_3_	1.53
CaO	10.07
MgO	4.60
MnO	0.05
Na_2_O	1.30
K_2_O	2.51
TiO_2_	0.25
P_2_O_5_	1.55
LOI	2.21

**Table 5:** National Highway Authority (NHA) class “A” materials selection criteria.

Mix Designation		NHA Class A
Compacted thickness		50–80 mm
mm	in	Percent Passing by Weight
25	1	100
19	3/4	90–100
13	1/2	–
10	3/8	56–70
5	No. 4	35–50
2	No. 8	23–35
1	No. 16	5–12
0	No. 200	2–8
**Asphalt Content by Weight**		
Percentage of total mix		3.5 (Min)

**Table 6:** NHA specifications for Marshall Parameters.

Description	NHA General Specification, 1998
Compaction blows	75
Stability (kg)	1000 (Minimum)
Flow (0.01 inch)	8–14
VMA	>13
VFB	70–90

**Table 7:** Determination of Effective Bitumen Content (EBC).

Parameter	% Bitumen Content
Bitumen content at the highest stability	4.5%
Bitumen content at the highest value of bulk specific gravity	4.5%
Bitumen content at the median of allowed percentages of air voids	5.0%
Effective Bitumen Content (EBC)—Mean Value	4.7%

## Data Availability

The data presented in this study are available on request from the corresponding author.
